# Preventing Postpartum Smoking Relapse Among Inner City Women: Development of a Theory-Based and Evidence-Guided Text Messaging Intervention

**DOI:** 10.2196/resprot.3059

**Published:** 2014-04-03

**Authors:** Kuang-Yi Wen, Suzanne M Miller, Linda Kilby, Linda Fleisher, Tanisha D Belton, Gem Roy, Enrique Hernandez

**Affiliations:** ^1^Fox Chase Cancer Center/Temple University Health SystemCancer Prevention and ControlPhiladelphia, PAUnited States; ^2^Manager of the Women, Infants and Children (WIC) Program in PhiladelphiaPhiladelphia, PAUnited States; ^3^The Center for Injury Research and PreventionThe Children's Hospital of PhiladelphiaPhiladelphia, PAUnited States; ^4^Temple University School of MedicineDepartment of Obstetrics, Gynecology, and Reproductive SciencesPhiladelphia, PAUnited States

**Keywords:** smoking relapse intervention, low-income women, mHealth, text messaging

## Abstract

**Background:**

Underserved women are at high risk for smoking relapse after childbirth due to their unique socioeconomic and postpartum stressors and barriers. Mobile text messaging technology allows delivery of relapse prevention programs targeted to their personal needs over time.

**Objective:**

To describe the development of a social-cognitive theory-based and evidence-guided text messaging intervention for preventing postpartum smoking relapse among inner city women.

**Methods:**

Guided by the cognitive-social health information processing framework, user-centered design, and health communication best practices, the intervention was developed through a systematic process that included needs assessment, followed by an iterative cycling through message drafting, health literacy evaluation and rewriting, review by target community members and a scientific advisory panel, and message revision, concluding with usability testing.

**Results:**

All message content was theory-grounded, derived by needs assessment analysis and evidence-based materials, reviewed and revised by the target population, health literacy experts, and scientific advisors. The final program, “Txt2Commit,” was developed as a fully automated system, designed to deliver 3 proactive messages per day for a 1-month postpartum smoking relapse intervention, with crave and lapse user-initiated message functions available when needed.

**Conclusions:**

The developmental process suggests that the application of theory and best practices in the design of text messaging smoking cessation interventions is not only feasible but necessary for ensuring that the interventions are evidence based and user-centered.

## Introduction

Tobacco use has remained the single most important modifiable cause of adverse pregnancy outcomes in the United States. Smoking accounts for 30% of deliveries of infants with low birth weights, 8% of preterm births, and 5% of all perinatal deaths [1-3]. Smoking during pregnancy and postpartum contributes to sudden infant death syndrome, changes in brain and nervous system development, and increased risk for infant ear and respiratory infections, in addition to cognitive and behavioral deficits [4,5]. Motivational factors during pregnancy, including protection of the health of the unborn baby, social pressure, pregnancy nausea, and loss of taste for tobacco, often result in a “suspension of smoking behavior” [6-9]. However, many women relapse after giving birth, suggesting that their commitment to cessation is not permanent. Indeed, approximately 45% resume smoking after only 3 months and up to 80% relapse within 1 year [10]. Low-income women are more likely than their higher income counterparts to smoke during pregnancy and relapse after childbirth. Therefore, they represent an important target population for postpartum relapse prevention interventions, especially given that they are typically difficult to access and contact [11].

Although there are programs designed to motivate underserved women who are at increased risk for smoking relapse [12,13], underserved women are less likely to successfully participate in and/or complete a smoking cessation program in the first place [14,15]. Conflicts in scheduling, lack of transportation, and family priorities are barriers to attending smoking cessation interventions [15,16]. These personal barriers are compounded by postpartum responsibilities and roles of the new mother, including childcare burden, relationship issues, medical problems, coping with maternal weight gain, weaning issues, and attempts to return to a nonpregnant state that can trigger a return to smoking [17-22]. In general, few interventions focus on the critical transition from pregnancy to the postpartum period, where the focus shiftsfrom the time-limited barriers that undermine protecting the fetus’s health to the psychosocial barriers that undermine the ongoing protection ofthe woman’s and new infant’s health [23,24]. Thus, there is a need for smoking relapse prevention interventions that are accessible to the target population and tailored to their specific psychosocial needs.

A key barrier that emerges for the delivery of evidence-based postpartum programs is access to the new mother.Encouragingly, due to the availability of modern technologies, one particularly well suited but under-utilized technology involves the use of mobile telephones to deliver health-related information to these hard-to-reach populations. Currently 90% of Americans own and use a mobile telephone with text messaging service and 47% of Americans use mobile phones to gain access to information on a daily basis [25]. Because mobile phones are also readily available to underserved women, text-based delivered interventions may benefit those who are most vulnerable and least readily accessible through alternative channels by providing intervention support and advice in a feasible and timely fashion [26]. Indeed, a recent Pew Research Center study found that African Americans and Latinos use mobile phones more than their white counterparts [27]. Furthermore, reading and sending text messages on a mobile phone are the most frequently engaged in activities [27]. In addition, mobile text messaging programs can be personalized, cost-effective, easily disseminated, and provide the anonymity that many people prefer [28,29]. Given that text messaging is available on all mobile phone platforms and with all providers, this technology demonstrates strong potential as a resource for behavior change interventions [29].

One successful example of using a text messaging program for pregnant women is the “Text4baby” program, which sends targeted health messages to promote healthy pregnancies and healthy babies [30]. Furthermore, studies show that text messages tailored to smoking habits and barriers to quitting have high usage, encourage quit attempts, and support smokers’ coping with crave and relapse [28,31-33]. Yet, to our knowledge, no study has examined text messaging as an intervention channel for delivering postpartum smoking cessation interventions [34]. In fact, the number of interventions specifically designed to prevent postpartum smoking relapse is limited, and only a few have focused on underserved minority populations [35-38]. Despite the growing body of literature reporting the positive outcomes of text messaging-based interventions for behavior change, including smoking cessation [31,34,39-42], there is a gap in the literature related to the application and systematic description of the developmental steps that need to be undertaken to produce and test the feasibility of text messaging interventions [43,44], a critical component of producing and disseminating a successful behavior change intervention [45,46]. To fill this void, guided by our research team’s cognitive-social health information processing (C-SHIP) model and health communication best practice principles, this paper describes an iterative user-centered developmental process of an evidence-based text messaging intervention designed to preventing smoking relapse among underserved inner city postpartum women.

## Methods

### Overview

With use of a user-centered approach, the intervention development followed a series of iterative steps to ensure that all content was understandable and evidence-based. Guided by the C-SHIP framework and health communication best practices, the text messaging intervention was developed through a systematic process that included needs assessment interviews, followed by an iterative cycling through text message drafting, revision through health literacy evaluation, review by target community interviews via cognitive response interviews, scientific advisory panel evaluation and feedback, and message revision, followed in turn by usability testing of the entire program.

The order of the development processes was established from our research team’s multimedia intervention development experience [47,48] and the relevant literature [43,49] to ensure the integration of formative research, pretesting content, pilot testing,and the involvement of stakeholders. The following sections detail each phase of the developmental process. The final intervention product (Txt2commit) is also described. This study was approved by the Institutional Review Board at Fox Chase Cancer Center.

### The Cognitive-Social Health Information Processing Model

Constructs from the C-SHIP model were used to guide the intervention development strategy and content. The C-SHIP model offers a unifying theoretical framework for assessing and addressing the psychosocial relapse factors typically experienced by postpartum women [50-52]. The model is built on cumulative findings and theorizing from diverse relevant sub-areas of cognitive-behavioral science and evidence-based psychosocial interventions, and integrates key cognitive and affective processes. Guided by this model, we identified 5 areas of psychosocial relapse risk factors among low-income, minority women: (1) low knowledge and perceived risk for relapse, (2) inadequate decisional balance (low pros and high cons of cessation), (3) high affect (distress), (4) negative beliefs (low self-efficacy and fatalistic beliefs), and (5) lack of self-regulatory, practical, and social support strategies. Over the past decade, a sizable literature has accumulated to support the role of these factors in undermining smoking behavior, acting as barriers of self-initiated cessation and enrollment in smoking programs, and decreasing quit rates and maintenance of smoking abstinence [18,53-56]

### Phase 1: Needs Assessment Individual Interviews

Participants (N=30) were recruited via flyers and staff referrals at 3 Women, Infants, and Children (WIC) clinics located in the inner city Philadelphia region. Interested women were invited to participate if they: (1) were 18 years of age or older, (2) had quit smoking for at least 1 pregnancy, and (3) had given birth to at least 1 child in the past 3 years. Interviews lasted approximately 20 minutes and included specific questions addressing the experiences, motivators, and techniques that had helped women maintain smoking abstinence after pregnancy, as well as personal, social, and environmental factors that triggered a postpartum relapse. Their mobile phone and text message use and preferences were also obtained. Upon completion of the interview, participants received a $20 gift card as compensation for their time and input.

Following an initial review of the interview transcripts, the research team developed a coding scheme based on the C-SHIP model. Responses categorized under C-SHIP were coded into 1 of the 5 psychosocial relapse risk factors. Subcategories in each domain were established as relevant themes emerged from the data. The intention behind coding responses into the C-SHIP processes was to systematically assess the major psychosocial relapse risk factors and identify the unique pattern of challenges faced by low-income, inner city women during the postpartum period.This information was used to guide the development of the Txt2commit intervention text message content.

### Phase 2: Text Message Content Development

As reported above, text message content development was grounded in the C-SHIP theoretical model and guided by a formative research approach through a needs assessment interview phase with the target population. In addition, we conducted a comprehensive literature review, as well as an evidence-based guideline search for the potential adoption of existing materials to adapt to the target population. Content component constructs included factors such as messages for increasing awareness of the risks associated with smoking on infant’s health (knowledge), techniques for controlling exposure to stressful cues that trigger the urge to lapse/relapse (affective distress), reinforcing beliefs about the woman’s ability to sustain smoking absence (self-efficacy), considering the consequences of continued smoking (decisional balance), and identifying new behaviors to be substituted for smoking-related activities (self-regulatory skills). To respond effectively to situations in which the participant required an instant message to deal with craving or lapse situations, text messages were developed to offer strategies and emotional support to cope with craving/lapse situations and strategies for how to get back on track.

### Phase 3: Health Literacy Evaluation

To ensure that the messages were designed for a low health-literate audience, 2 health literacy experts systematically evaluated all drafted messages using software and their health literacy expertise. The program Health Literacy Advisor (Health Literacy Innovations, LLC) was used to scan and highlight the text for complex terms, complex health terms, polysyllabic words (ie, words with more than 3 syllables), and long sentences (ie, sentences with 12 or more words).The findings from the health literacy analysis were then evaluated by the 2 health literacy experts and the research team to further revise the text messages to improve readability.

### Phase 4: Message Review Cognitive Response Interviews

Following the revisions for health literacy, the messages were reviewed and vetted by participants (N=30) recruited at the 3 WIC offices with the same eligibility criteria identical to those of the needs assessment phase.Each participant reviewed 21 messages and was asked to think aloud about the messages, paraphrase the content, and respond to other inquiries and probes using the cognitive response technique [57]. Specifically, participants were asked if messages were relevant to them, understandable, and helpful in a stressful situation, as well as whether and how they would change the wording of the messages. Additionally, they rated each message from 1 (very poor) to 5 (very good) based on how helpful the message would be if they were inclined to smoke. Upon completion of the interview, participants received a $20 gift card as compensation for their time and input. The interview data and field notes were reviewed by the research team and messages were subsequently revised according to the participants’ ratings and comments through a consensus process. Low-rated messages were flagged for possible removal.

### Phase 5: Text Message Scientific Advisory Panel Review

A multidisciplinary scientific advisory panel was convened to provide further insight into the cultural sensitivity, appropriateness, and message appeal of the intervention for the target population. The panel (N=7) was composed of smoking cessation professionals, with expertise in psychosocial behavior, health communication, WIC management, and community perspectives. Panel members were provided with a list of messages (with information on revisions made in the health literacy evaluation process and suggestions made by participants), and the C-SHIP coding guide for each message. Members of the advisory panel evaluated the appropriateness of the content of each of the messages and provided their recommended revisions to the wording and readability of each message from their individual perspective and expertise. The research team then reviewed the advisors’ comments and made necessary modifications to produce the final program.

### Phase 6: 1-Week Usability Testing

Ten participants, with the same eligibility criteria identical to those of the needs assessment phase, were recruited to pretest the program for usability testing following the scientific advisory review phase. The program was delivered over 1 week. Participants were provided with phones equipped with text messaging service and were instructed as follows: complete (open and confirm receipt of) 7 system-initiated messages (1 per day), and initiate 3 crave (described below) and 3 lapse message requests (described below) during the 1-week testing phase. Participants were debriefed individually by research staff at the conclusion of the usability testing and received a $20 gift card as compensation for their time and input. They were asked to provide the following overall feedback: system-initiated text message understandability, cultural appropriateness of text message wording, ease of use of crave and lapse functions, and problems encountered in use of the program. Each participant was also asked to provide additional comments concerning the intrusiveness, timing, and general burden imposed by using the program. Feedback was compiled and evaluated by the research team to determine whether adjustments in the messages, system, or study procedures warranted correction or adjustment, with subsequent implementation of necessary corrections and adjustments.

## Results

### Phase 1: Needs Assessment Interviews

The findings of the needs assessment interviews are reported elsewhere; participant characteristic information is presented in Table 1. In summary, participants (N=30) expressed the following reasons for why they resumed or refrained from smoking following childbirth: (1) motherhood demands (26/30, 87%), (2) partner and family relationships (22/30, 73%), and (3) the presence of other smokers in the environment (15/30, 50%). Participants reported 4 main strategies that helped them cope successfullywith postpartum cravings and relapses, including being informed of smoking risks (26/30, 86%), maintaining goal-oriented thoughts (18/30, 60%), thinking about the pros of quitting (26/30, 87%), and receiving positive social support from families and friends (27/30, 90%).

With regard to phone habits, most participants (21/30, 70%) had a home phone, but even more were mobile phone users (25/30, 83%). In addition, 93% (28/30) of participants reported that they used their mobile phones for texting, and 83% (25/30) reported that they texted on a daily basis. However, none of the participants in this population owned a smartphone. Therefore, our intervention only used text-messaging delivery, which is compatible with all models of mobile phones. Furthermore, when inquiring about their attitudes toward text message styles for a smoking cessation/relapse program, a more formal tone of content without the use of common text messaging abbreviations was preferred because it was thought to increase the creditability of the program. Information gathered from the needs assessment interviews was synthesized by the research team to guide the drafting of the text message content.

### Phase 2: Message Development

A pool of 204 messages was initially developed based on the findings of the needs assessment and a review of the relevant literature.The emerging themes from the needs assessment content analysis were also integrated into the message drafting. The C-SHIP model provided a theoretical guide for message creation because this model indicates specific areas that are especially relevant for health interventions. Smoking facts from several evidence-based resources, including the National Cancer Institute’s fact sheet concerning the harms of smoking, were identified and extracted to be included within the messages where appropriate.

The text messaging intervention developed included 3 main components: low-frequency messages to be sent during the third trimester (1 per week for engagement before the start of the main intervention), high-frequency messages to be sent after participants had given birth (1 message per day for 1 month), and messages available upon participant request when experiencing a craving for a cigarette or a lapse into having smoked one. The messages were systematically and comprehensively developed to address each component, so that some messages were specific to the prenatal or postpartum period and some to the situation of craving or lapsing. The drafted messages were coded across the 5 C-SHIP constructs by the research team.

### Phase 3: Health Literacy Review

The initial assessment of the 204 text messages identified a number of issues, including the use of polysyllabic words, complex health terms, complex nonmedical terms, and long sentences. After the 2 health literacy experts reviewed and modified each message in collaboration with the research team, a second assessment using Health Literacy Advisor revealed a significant reduction in the following health literacy problems: a 72% reduction in polysyllabic words, 79% reduction in complex health terms, 91% reduction in complex nonmedical terms, and 47% reduction in long sentences. Most of the complex terms remaining in the text messages were words that pertained to the message content and the study, such as tobacco, smoking, and cancer. During the message review interview phase described below, these words were specifically tested and found to be readily recognized by study participants, indicating that they were not difficult to read or understand.

### Phase 4: Message Review Cognitive Response Interviews

**Figure 1:**
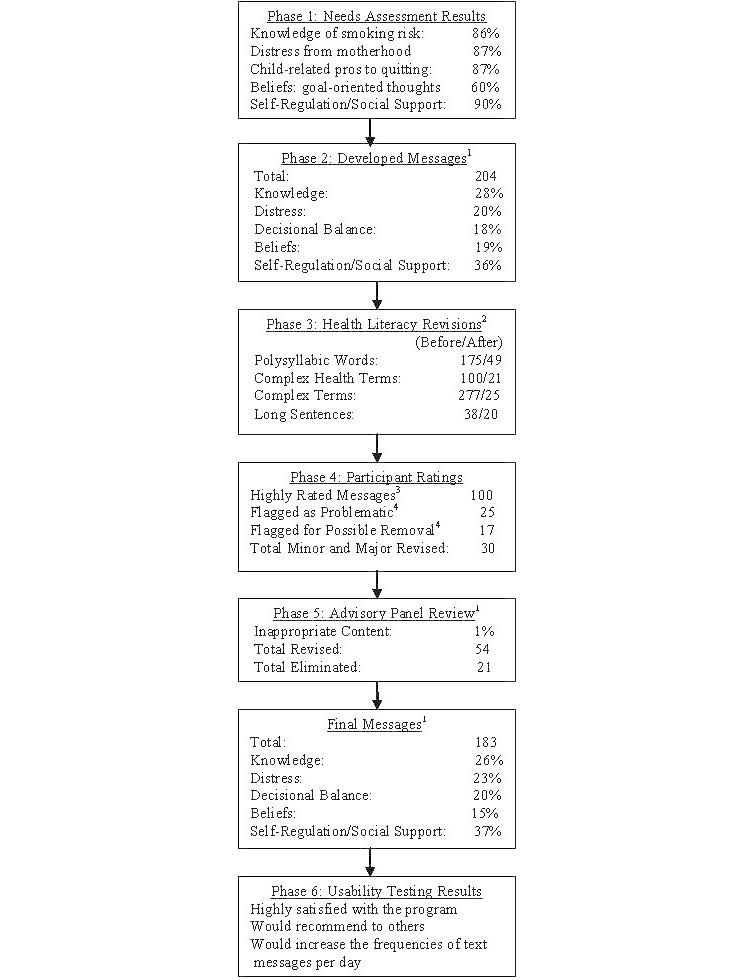
Steps and results from each development phase. ^1^Messages may fall under multiple categories; thus, percentages do not sum to 100%. ^2^Eleven messages required additional revision to fit within the 159-character limit for text messages. ^3^Highly rated messages received a mean rating over 4.5 (out of 5) for “Overall Helpful” and were found to be “Helpful If Stressed” by all raters. ^4^Messages were flagged if they received a mean rating less than 4 (out of 5) for “Overall Helpful”; these were then identified for possible removal if two-thirds or more of raters found it “Not Helpful If Stressed,” or designated “Problematic” in all other cases.

Messages were reviewed by 30 women at 3 different WIC clinic sites using the cognitive response interview technique. Table 1 displays the sample characteristics. Participants provided specific comments regarding improvements that could be made to the messages, as well as their general reaction to each message. This feedback was used to guide message revisions. For all but 1message, at least 67% (20/30) of participants found the messages to be understandable. Messages were generally viewed as helpful, obtaining a mean rating of 4.2 on a 5-point scale, and 50% of the messages were highly rated (>4.5). However, 25 messages received lower ratings, as summarized in Figure 1.These low-rated messages were subsequently either revised (n=8) or eliminated (n=17). We also received universal agreement from the participants that 2-3 messages per day would be appropriate during the postpartum period.

**Table 1:** Background variables for needs assessment and message review interview sample (N=30).

Variable		Needs assessment n (%)	Message review n (%)
**Race/ethnicity**			
	Hispanic	6 (20)	6 (20)
	White	11 (37)	4 (13.3)
	African American	12 (40)	19 (63.3)
	Other	1 (3.3)	1 (3.3)
**Marital status**			
	Single	28 (93.3)	23 (76.7)
	Married/cohabiting	2 (6.7)	4 (13.3)
	Separated	0 (0)	2 (6.7)
	Divorced	0 (0)	1 (3.3)
**Income**			
	$0-15,000	17 (56.7)	18 (60)
	$15,000-30,000	10 (33.3)	11 (36.7)
	$30,000-45,000	3 (10)	0 (0)
	$45,000-60,000	0 (0)	1 (3.3)
**Education**			
	8-11 years	4 (13.3)	5 (16.7)
	H.S. grad/GED	14 (46.7)	16 (53.3)
	Vocational/tech	4 (13.3)	2 (6.7)
	Some college	6 (20)	7 (23.3)
	Bachelor’s degree	2 (6.7)	0 (0)
**Health insurance**			
	Medical assistance	28 (93.3)	24 (80)
	None	2 (6.7)	6 (20)
Employed		6 (20)	10 (33.3)
Home phone owner		21 (70)	14 (46.7)
Cell phone owner		25 (83.3)	25 (83.3)
**Smoking frequency**			
	Every day	17 (56.7)	12 (40)
	Some days	5 (16.7)	6 (20)
	None	8 (26.7)	12 (40)

### Phase 5: Text Message Scientific Advisory Panel Review

All messages were evaluated by the advisory panel in relation to the relevance of the messages to the population, as well as their coverage of factors involved in the C-SHIP framework. A final review of the advisory panel input by the research team resulted in slight modifications to 50% of the messages and 21 additional messages being eliminated.Table 2 provides examples of the message revision process and Table 3 provides samples of final text messages corresponding to the 5 C-SHIP constructs. For most messages, the basic content of the original message was retained, but revisions improved the readability and specificity of the message.

**Table 2:** Examples of message revision process.

Original message	Health literacy revision	Participant comment	Comment revision	After advisory panel
Research shows that female smokers lose an average of 14.5 years off their lives because of smoking.	Research shows that women who smoke lose about 14.5 years off their lives.	“It’s confusing. . . [when] it says this is about 14.5 years off their lives.”	Research shows that women who smoke live about 14.5 years less than those who don't.	Women who don't smoke live about 15 years more than women who do smoke.
The American Lung Association reports that smoking during pregnancy accounts for 10% of all infant deaths.	The American Lung Association reports that smoking during pregnancy is the reason for 10% of all infant deaths.	"Good message. Gives people info about the risks of smoking. It makes pregnant moms think about their unborn child."	None	The American Lung Association reports that smoking during pregnancy is the reason for 10% of all infant deaths.
Think about it. Would you rather feel tired and less energized, or healthy and rejuvenated? You're on the right path to a healthier you!	Think about it. Would you rather feel tired and have less energy, or healthy and rejuvenated? You're on the right path to a healthier you!	None	Would you rather feel tired and have less energy because you smoke? Or feel healthy and rejuvenated as a nonsmoker? Stay on the right path to a healthier you!	By not smoking, you will feel healthy and fresh instead of tired and drained. Stay on the right path to a healthier you!

**Table 3:** Samples of final text messages across 5 C-SHIP constructs.^a^

Knowledge	Decisional balance	Distress	Beliefs	Self-regulation /social support
Kids whose parents smoke get bronchitis and pneumonia much more often.	Caring for a baby takes a lot of energy! If you do not smoke, you will feel more active during the day to play with your baby.	Feeling extra stressed and grouchy today? Try playing soft music to help you relax. It may help calm any sudden urges and relax you and your fussy baby.	Smoking while pregnant puts your baby at higher risk for ear infections and asthma attacks. Be glad you chose to quit!	Put away ashtrays, matches, and lighters. Trade them for things that remind you not to smoke, like a list of reasons for quitting, or a family photo.
With each cigarette, you breathe in more than 4000 chemicals. Don’t be fooled, all forms of tobacco can hurt you and your baby.	If you hope to have more children, now is the time to quit. Smoking raises your chance of having trouble getting pregnant again.	It is normal to feel stressed with a new baby in the household. Remember to make “me” time!	At the end of each day, think about how you didn't smoke at all. Be thankful for that moment. It will make your thoughts of success stronger.	Make sure your car and home are smoke free. Do it for yourself and others, especially for your baby.
Babies whose mothers smoked during pregnancy are up to three times as likely to die from sudden infant death syndrome (SIDS) as babies of nonsmokers.	Not smoking makes you a better role model for your kids. A child raised by a nonsmoker is less likely to start smoking. Stay smoke free for your baby.	Do not worry if you are gaining a few pounds. Weight gain is normal when you stop smoking. Keep a healthy diet and talk to your doctor if you are concerned.	You quit smoking for a reason. Think about this reason often. It will never be less true, but it could become less important if you forget about it.	People who lift weights have spotters. You too should have a spotter! Pick someone you trust to support you and help you to stay smoke free.

^a^Some messages may fall under multiple categories.

### Phase 6: 1-Week Usability Testing

Ten participants were recruited from the same 3 WIC clinic sites as in the interview phases to participate in a 1-week usability testing session. Each participant was provided with a mobile phone and instructions for mobile phone and message use. Each participant received 7 system-initiated messages over a 1-week period and was instructed to initiate 3 crave and 3 lapse message requests. Among the 10 enrolled participants, 8 successfully completed the testing and responded to the evaluation interview. All 8 participants identified the messages as helpful and reported being satisfied with the messages, as well as with the crave and lapse functions. They all stated that they would use the program and recommend it to others. They also suggested changes, including making the system-initiated messages more frequent (3 per day) and adding voice and video messages to the program. Based on participants’ feedback, the research team finalized the “Txt2commit” program, which provided: (1) 1 text message per week during the prenatal period (27 weeks gestation and on) to enhance and sustain program engagement, (2) 3 text messages daily during the first postpartum month, and (3) crave and lapse functions so that participants could request additional messages when feeling the need. The final program was tested in a larger feasibility pilot study, results of which will be reported elsewhere for ease of communication. Figure 1 provides an illustration of the order and findings throughout different phases of the development, including the changes of the numbers of messages over time.

## Discussion

The development of the Txt2commit mobile text messaging intervention relied on a combination of theory and evidence, systematic attention to health literacy issues, and integration of a user-centered approach throughout the developmental process. This research adds to the emerging literature supporting the critical role of a comprehensive strategy for the design of mHealth interventions and highlights the value of a scientifically based health literacy focus. This study aimed to stimulate discussion among target users about how to use mobile text messaging as a platform for delivering smoking cessation interventions for underserved postpartum women, as well as demonstrate a rigorous process of development to ensure that the messages are potent, easily understood, and delivered in an effective manner. Using an iterative developmental process, a multidisciplinary team, and user input, the text messaging intervention was progressively developed and refined based on formative participatory evaluations to achieve a culturally—and linguistically—appropriate psychosocial program that provides relevant content to underserved women who quit smoking during their pregnancies.

The needs assessment, message review, and cognitive response interviews were all essential to identify gaps in women’s needs in postpartum and smoking relapse trigger stressors, which resulted in relevant and appropriate text messaging content and format. Furthermore, the health literacy review and painstaking editing significantly increased the readability and accessibility of the information. The final usability testing confirmed the preferred frequency of message delivery and the usefulness of the crave and lapse functions, prior to the final production and pilot study implementation. The intensity and functionalities of our final intervention program design is comparable to most of the studies in the text messaging smoking cessation literature, with an average of 1-5 text messages per day and the availabilities of crave and lapse functions [28,58,59].

As technology advances become more common as a delivery mechanism for health behavior interventions, it is important to recognize that the impact will depend on the interdigitation of well-designed content, technology, and end user input.Given advances in the field of behavior medicine, it has become increasingly clear that knowledge alone does not change behavior. Likewise, technology alone does not change behavior, but the combination of theory-based best practice approaches in content development, coupled with technology, increases access and reach and has great promise for improving outcomes through attention to evidence-based cessation barriers. The strengths of the study include the use of an established C-SHIP theoretical framework, application of health communication and health literacy best practices to guide text messaging content drafting and revision, extensive input from a community-based sample at various steps in development, and the use of a multidisciplinary expert advisory board. This approach extends our prior research, where the C-SHIP framework was successfully applied to smoking cessation and multimedia intervention behavior change studies [47,50,52,60,61]. To our knowledge, this is the first text messaging intervention guided by the C-SHIP framework. Because research has shown that messaging interventions designed by using behavioral theory are more likely to be successful [29], this paper contributes to the field by demonstrating how to apply a well-established framework in the context of new technology delivery. We also found that using a guiding theoretical framework, coupled with input from a diverse group of stakeholders, was helpful during the message revision process, particularly in terms of better addressing the target conceptual construct contained in each message as well as keeping the message within the limit of 160 characters.

This study provides insight into the systematic development of text messaging interventions for use in other behavior change contexts. The prevalence of mobile phone use and text messaging among the present underserved sample indicates the potential of text messaging as a delivery modality in a variety of health settings and behaviors. Therefore, the development concepts used in this intervention should be applicable to the development process for other health-related mHealth interventions, especially those targeted to diverse underserved populations. One important lesson learned was that although text messaging was an acceptable channel to communicate smoking relapse messages, postpartum women indicated their preference that these messages be written using formal language to highlight the credibility of the information.

As the vision of eHealth/mHealth is beginning to be actualized, development must also allow for the involvement of “consumers with diverse perspectives, circumstances, capacities, and experiences in the design of, evidence-based eHealth tools” [62]. This study supports the importance of a comprehensive developmental process as an essential foundational basis for rigorous research in this area [47-49,63]. The goal is to ensure that the intervention messages are appropriate, relevant, and understandable, with a view to developing meaningful interventions that can be evaluated for effectiveness, over the short and long term. With the increasing prevalence of mobile phone and text messaging use in underserved inner city populations in the United States and in low-income countries at the global level, harnessing this technological approach has great potential to address health disparities and improve health outcomes in a variety of challenging behavioral contexts.

## Abbreviations

C-SHIP: cognitive-social health information processing
WIC: women, infants, and children
